# Oral kanglaite injection (KLTI) attenuates the lung cancer-promoting effect of high-fat diet (HFD)-induced obesity

**DOI:** 10.18632/oncotarget.11212

**Published:** 2016-08-11

**Authors:** Ning Cao, Xiaofang Ma, Zhenzhen Guo, Yaqiu Zheng, Shengnan Geng, Mingjing Meng, Zhenhua Du, Haihong Lin, Yongjian Duan, Gangjun Du

**Affiliations:** ^1^ Institute of Pharmacy, Pharmacy College of Henan University, Jinming District, Kaifeng, Henan Province 475004, China; ^2^ Department of Oncology, The First Hospital Affiliated to Henan University, Kaifeng, Henan Province 475001, China

**Keywords:** kanglaite injection (KLTI), overweight and obesity, metabolic dysfunction, cellular signaling molecules, tissue edema

## Abstract

Obesity is a risk factor for cancer and cancer-related mortality, however, its role in lung cancer progression remains controversial. This study aimed to assess whether high-fat diet (HFD)-induced obesity promotes lung cancer progression and whether the promotion can be decreased by Kanglaite injection (KLTI). *In vivo*, HFD-induced overweight or obesity increases the lung carcinoma incidence and multiplicity in a urethane-induced lung carcinogenic model and cancer-related mortality in a LLC allograft model by increasing oxidative stress and cellular signaling molecules including JAK, STAT3, Akt, mTOR, NF-κB and cyclin D1. These changes resulted in increases in vascular disruption and the lung water content, thereby promoting lung epithelial proliferation and the epithelial-mesenchymal transition (EMT) during carcinogenesis. Chronic KLTI treatment substantially prevented the weight gain resulting from HFD consumption, thereby reversing the metabolic dysfunction-related physiological changes and reducing susceptibility to lung carcinogenesis. *In vitro*, KLTI significantly suppressed the proliferation and induced apoptosis and differentiation in 3T3-L1 preadipocyte cells and attenuated endothelial cell permeability in HUVECs. Our study indicates that there is a potential relationship between obesity and lung cancer. This is the first study to show that obesity can directly accelerate carcinogen-induced lung cancer progression and that KLTI can decrease the lung cancer-promoting effect of HFD-induced obesity.

## INTRODUCTION

Lung cancer is the leading cause of cancer death in both more and less developed countries [1], while China is experiencing more and more days of serious air pollution recently and has the highest lung cancer burden in the world [2]. Currently, the control of lung cancer has attracted worldwide attention, whereas focusing on prevention is an important factor to reduce the burden of screening, treatment, and lung cancer deaths [3]. However, much work is needed to determine whether these strategies will be viable. Studies on the relative risk factors of lung cancer have played an important role in its prevention. Recent epidemiological and experimental evidence indicates that obesity and its related metabolic abnormalities are associated with the development of certain epithelial malignancies [4]. There is also expanding evidence of the role of obesity in cancer development, treatment, and survival [5]. Lung cancer is not considered an obesity-related cancer, furthermore, obese patients with non-small cell lung cancer (NSCLC) were reported to have better survival outcomes compared to NSCLC patients of normal weight [6]. However, there is also a possible direct correlation with body mass index (BMI) that would support obesity as a potential risk factor for lung cancer. In fact, cancer patients who are obese are at a greater risk of recurrence with shorter disease-free survival rates than non-obese patients [7]. Although it has been shown enhanced spontaneous metastasis of Lewis lung carcinoma in C57BL/6 mice fed an high fat diet (HFD) [8, 9], there is no published information regarding whether obesity may affect carcinogen-induced lung cancer.

Kanglaite injection (KLTI) is a microemulsion of Coix seed oil extracted from Chinese medicine-Coix seed which has been used in China as an effective clinical drug for over a thousand years [10]. It contains numerous ingredients (0.1 g Coix seed oil per ml consisting of 20.8% 1,2,3-trioleylglycerol (C_57_H_104_O_6_), 19.2% 2,3-dioleoyl-1-linoleylglycerol (C_57_H_102_O_6_), 18.9% 1,2-dilinoleyl-3-oleylglycerol (C_57_H_100_O_6_), 14.8% 2-linoleyl-3-oleyl-1-palmitoylglycerol (C_55_H_100_O_6_), 11.8% 2,3-dioleyl-1-palmitoylglycerol (C_55_H_102_O_6_), 7.5% 1,2-dilinoleyl-3- palmitoylglycerol (C_55_H_98_O_6_) and 7.0% 1,2,3-trilinoleylglycerol (C_57_H_98_O_6_), Zhejiang Kanglaite Pharmaceutical Co. Ltd, China), has been proven effective in treating multiple cancers in China, and is also the first drug derived from a traditional Chinese herbal remedy approved by the USA Food and Drug Administration to undergo clinical trials in the United States [11]. A meta-analysis found that KLT injection in combination with chemotherapy was associated with improved response rate, quality of life, and symptoms, and a reduced incidence of adverse events compared with chemotherapy alone in patients with NSCLC [12]. The results from Liu et al also suggest that KLT injection combined with chemotherapy can improve the short-term efficacy, performance status and decrease the risk of gastrointestinal reaction compared with systematic chemotherapy alone in the treatment of advanced non-small cell lung carcinoma [13]. Although some studies have provided observational data on the efficacy of KLTI in cancer treatment, only few of them propose mechanisms that would permit a better understanding of the causal mechanisms of this effect [14]. Considering that Coix seed has been used as a dietary supplement and Coix seed oil could reduce the abdominal fat tissue [15], we hypothesized that HFD-induced obesity facilitates lung carcinoma formation and its pro-carcinogenic effect can be prevented by KLTI. The present experiments were conducted to test that hypothesis in mice fed a HFD or standard rodent chow (SRC) using a urethane-induced lung cancer model and a LLC allograft model, if so, tried to explore its mechanism.

## RESULTS

### HFD-induced overweight or obesity increases lung carcinoma formation and cancer-related mortality

**Figure 1:**
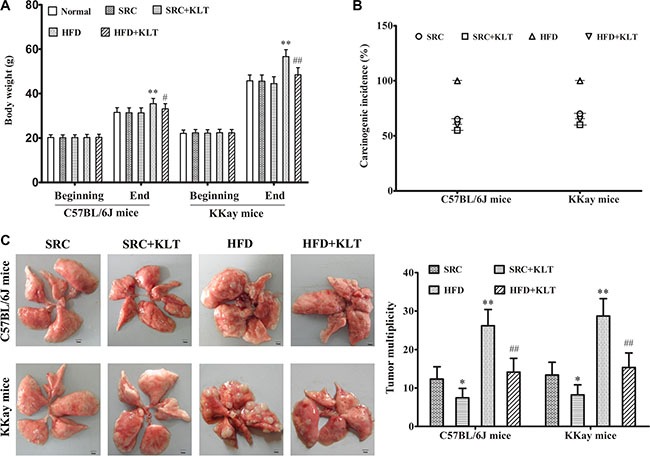
HFD-induced overweight or obesity, increases lung carcinoma incidence and multiplicity Mice received intraperitoneal injections of urethane once weekly for 10 weeks. Following these injections, the mice were fed the SRC or the HFD and were administered KLTI once daily for 20 weeks. (**A**) Body weight of mice fed a HFD or SRC. (**B**) Lung carcinoma incidence. (**C**) Tumor multiplicity. The data are presented as the mean ± SD, and statistical significance was determined by ANOVA, and unpaired, 2-tailed Student's *t* test was used to compare difference between two groups. *n* = 20, *vs SRC or ^#^*vs* HFD *P* < 0.05; **vs SRC or ^##^*vs* HFD *P* < 0.01. HFD, high-fat diet; SRC, standard rodent chow; KLTI, kanglaite injection. Normal was defined as the non-carcinogenic mice fed in parallel.

**Figure 2:**
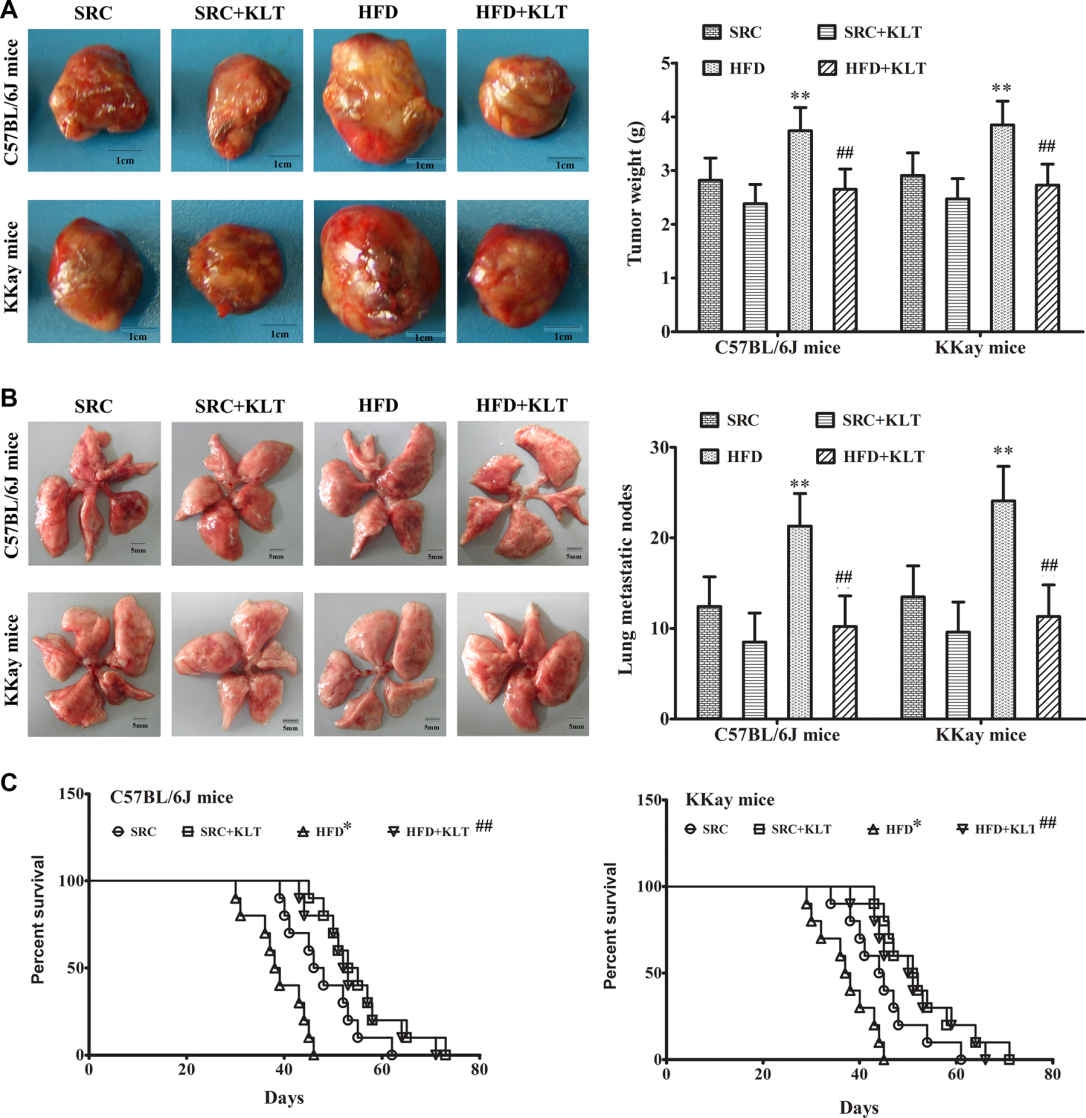
HFD-induced overweight or obesity decreases cancer-related survival LLC cells were injected subcutaneously into the lateral axilla of the mice. Following tumor inoculation, the mice were administered KLTI once daily for four weeks. (**A**) Tumor size. (**B**) The number of lung metastasis. (**C**) Survival curve. The data are presented as the mean ± SD, and statistical significance was determined by ANOVA, and unpaired, 2-tailed Student's *t* test was used to compare difference between two groups. Survival curves were constructed using the Kaplan–Meier method, and log-rank tests were performed to evaluate differences between groups. *n* = 10, *vs SRC or ^#^*vs* HFD, *P* < 0.05; **vs SRC or ^##^*vs* HFD, *P* < 0.01. HFD, high-fat diet; SRC, standard rodent chow; KLTI, kanglaite injection. Normal was defined as the non-carcinogenic mice fed in parallel.

To assess the potential relationship between obesity and lung cancer, two experimental diets and two lung cancer models were used. Urethane-induced lung cancer has a similar histological appearance and similar molecular changes as those in human lung adenocarcinoma [16]. This animal model has been optimised for manageable carcinogenesis and was used to assess pro-carcinogenic effect of HFD-induced overweight or obesity [17]. LLC allograft model is a suitable mouse model to evaluate therapeutic effects and was used to assess cancer-related mortality [18]. It was shown that the body weight (BW) was 35.5 ± 2.3 g in HFD-fed C57BL/6J mice (overweight, *P* < 0.05) *vs* 31.4 ± 2.2 g in SRC-fed C57BL/6J mice and was 56.7 ± 3.1 g in HFD-fed KKAy mice (obesity, *P* < 0.01) *vs* 45.6 ± 2.8 g in SRC-fed KKAy mice (Figure 1A). In addition, in a urethane-induced lung carcinogenesis model, the lung carcinoma incidence was 100% in HFD-fed C57BL/6J mice *vs* 65% in SRC-fed C57BL/6J mice and was 100% in HFD-fed KKAy mice *vs* 70% in SRC-fed KKAy mice. Multiplicity was 26.2 ± 4.2 in HFD-fed C57BL/6J mice (*P* < 0.01) *vs* 12.3 ± 3.2 in SRC-fed C57BL/6J mice and was 28.7 ± 4.5 in HFD- fed KKAy mice (*P* < 0.01) *vs* 13.4 ± 3.3 in SRC-fed KKAy mice (Figure 1B and 1C). Further, in a LLC allograft model, the tumor size was 3.74 ± 0.43 g in HFD-fed C57BL/6J mice (*P* < 0.01) *vs* 2.82 ± 0.41 g in SRC-fed C57BL/6J mice and was 3.85 ± 0.44 g in HFD-fed KKAy mice (*P* < 0.01) *vs* 2.91 ± 0.42 g in SRC-fed KKAy mice (Figure 2A) The number of lung metastasis was 21.3 ± 3.6 in HFD-fed C57BL/6J mice (*P* < 0.01) *vs* 12.4 ± 3.3 in SRC-fed C57BL/6J mice and was 24.1 ± 3.8 in HFD-fed KKAy mice (*P* < 0.01) *vs* 13.5 ± 3.4 in SRC-fed KKAy mice (Figure 2B). The median survival of cancer-related survival was 39 d in HFD-fed C57BL/6J mice (*P* < 0.05) *vs* 48 d in SRC-fed C57BL/6J mice and was 37 d in HFD-fed KKAy mice (*P* < 0.05) *vs* 45 d in SRC-fed KKAy mice (Figure 2C). However, no significant differences in lung carcinogenesis or tumor development were observed between the two strains of the two lung cancer models following consumption of the same diet (Figures 1B–1D and 2A–2C), indicating the important role of diet in lung cancer.

### HFD-induced overweight or obesity leads to energy imbalance

**Figure 3:**
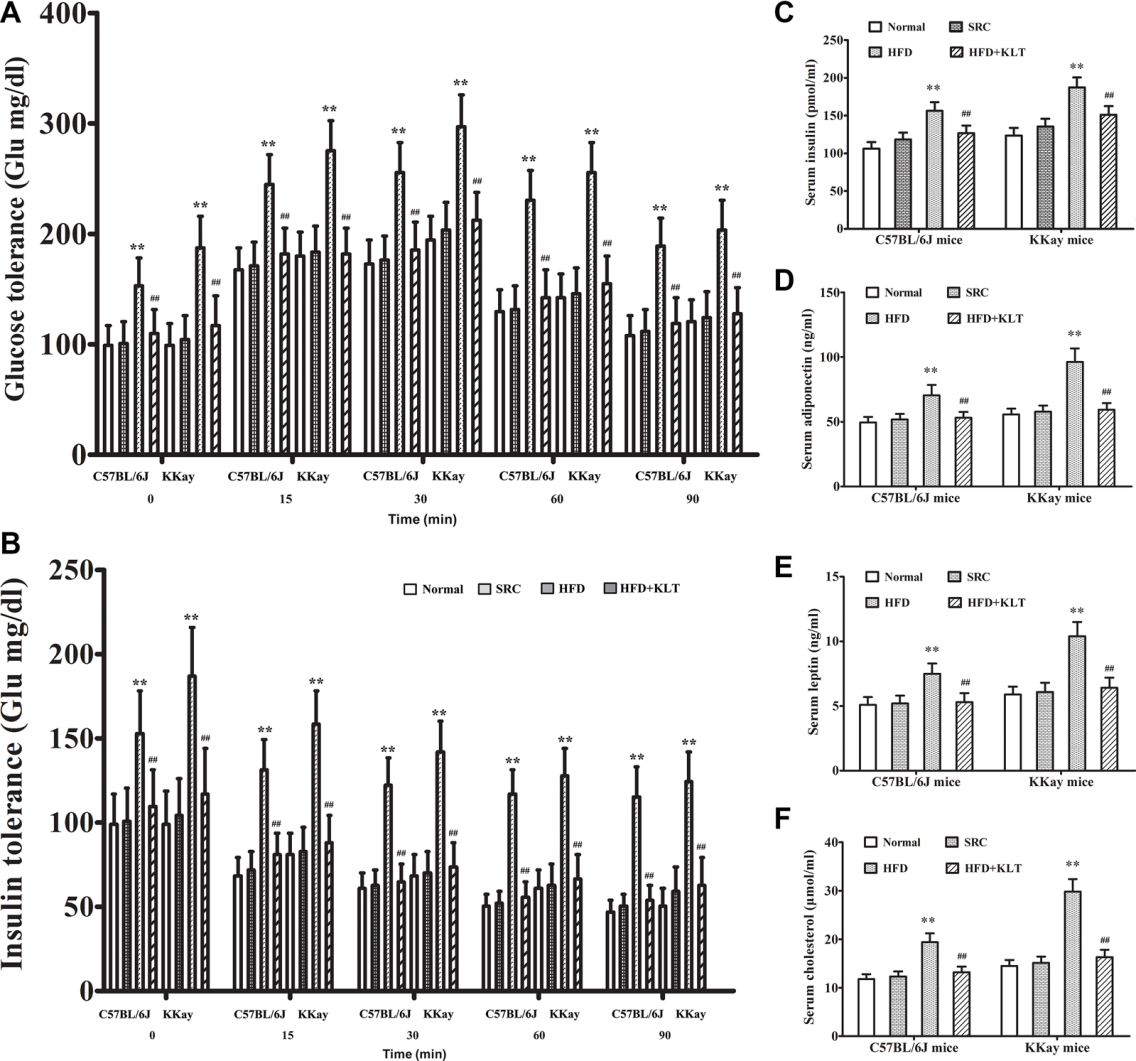
HFD-induced overweight or obesity leads to energy imbalance (**A**) Glucose tolerance. (**B**) Insulin tolerance. (**C**) Serum insulin. (**D**) Serum adiponectin. (**E**) Serum leptin. (**F**) Serum cholesterol. The data are presented as the mean ± SD, and statistical significance was determined by ANOVA, and unpaired, 2-tailed Student's *t* test was used to compare difference between two groups. *n* = 5, **vs SRC or ^##^*vs* HFD; *P* < 0.01. HFD, high-fat diet; SRC, standard rodent chow; KLTI, kanglaite injection. Normal was defined as the non-carcinogenic mice fed in parallel.

**Figure 4:**
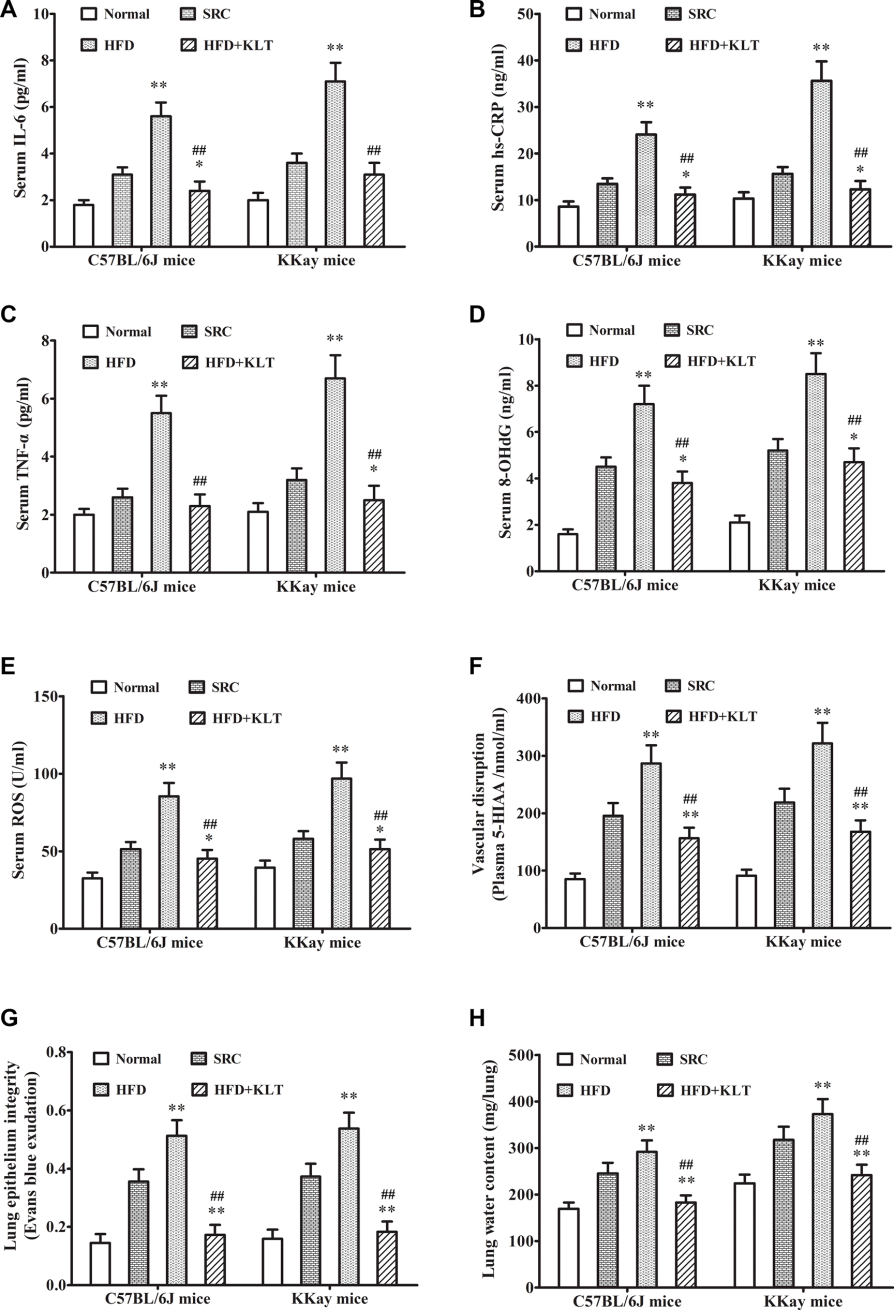
HFD-induced overweight or obesity leads to inflammation and tissue edema (**A**) Serum IL-6. (**B**) Serum hs-CRP. (**C**) Serum TNF-α. (**D**) Serum 8-OHdG (**E**) Sertum ROS. (**F**) Plasma 5-HIAA. (**G**) Lung epithelial integrity. (**H**) Lung water content. The data are presented as the mean ± SD, and statistical significance was determined by ANOVA, and unpaired, 2-tailed Student's *t* test was used to compare difference between two groups. *n* = 5, *vs SRC, *P* < 0.05; **vs SRC or ^##^*vs* HFD, *P* < 0.01. HFD, high-fat diet; SRC, standard rodent chow; KLTI, kanglaite injection. Normal was defined as the non-carcinogenic mice fed in parallel.

A pathologic feature of overweight or obesity is involved in the overall state of energy imbalance [19]. To assess this feature, we performed insulin and glucose tolerance tests, a clinical chemistry test and oxidative stress analysis at 21 week point after induction in a urethane-induced lung carcinogenic model. Compared to the same strain of mice fed the SRC, the overweight or obese mice exhibited significantly lower insulin and glucose tolerances (Figure 3A and 3B). Consistent with these decreased tolerances, serum insulin was 156.3 ± 11.4 pmol/ml in HFD-fed C57BL/6J mice (*P* < 0.01) vs 118.2 ± 9.1 pmol/ml in SRC-fed C57BL/6J mice and was 187.5 ± 13.2 pmol/ml in HFD- fed KKAy mice (*P* < 0.01) vs 135.4 ± 10.6 pmol/ml in SRC-fed KKAy mice (Figure 3C). Serum adiponectin was 70.4 ± 8.2 ng/ml in HFD- fed C57BL/6J mice (*P* < 0.01) vs 51.8 ± 4.3 ng/ml in SRC-fed C57BL/6J mice and was 96.3 ± 10.5 ng/ml in HFD- fed KKAy mice (*P* < 0.01) vs 57.9 ± 4.6 ng/ml in SRC-fed KKAy mice (Figure 3D). Serum leptin was 7.5 ± 0.8 ng/ml in HFD- fed C57BL/6J mice (*P* < 0.01) vs 5.2 ± 0.6 ng/ml in SRC-fed C57BL/6J mice and was 10.4 ± 1.1 ng/ml in HFD- fed KKAy mice (*P* < 0.01) vs 6.1 ± 0.7 ng/ml in SRC-fed KKAy mice (Figure 3E). Serum cholesterol was 19.4 ± 1.8 μmol/ml in HFD- fed C57BL/6J mice (*P* < 0.01) vs 12.3 ± 1.1 μmol/ml in SRC-fed C57BL/6J mice and was 29.8 ± 2.6 μmol/ml in HFD- fed KKAy mice (*P* < 0.01) vs 15.1 ± 1.3 μmol/ml in SRC-fed KKAy mice (Figure 3F). Similarly, consistent with previous reports, serum IL-6 was 5.6 ± 0.6 pg/ml in HFD- fed C57BL/6J mice (*P* < 0.01) vs 3.1 ± 0.3 pg/ml in SRC-fed C57BL/6J mice and was 7.1 ± 0.8 pg/ml in HFD- fed KKAy mice (*P* < 0.01) vs 3.6 ± 0.4 pg/ml in SRC-fed KKAy mice (Figure 4A). Serum hs-CRP was 24.1 ± 2.6 ng/ml in HFD- fed C57BL/6J mice (*P* < 0.01) vs 13.5 ± 1.2 ng/ml in SRC-fed C57BL/6J mice and was 35.6 ± 4.2 ng/ml in HFD- fed KKAy mice (*P* < 0.01) vs 15.6 ± 1.5 ng/ml in SRC-fed KKAy mice (Figure 4B). Serum TNF-α was 5.5 ± 0.6 pg/ml in HFD- fed C57BL/6J mice (*P* < 0.01) vs 2.6 ± 0.3 pg/ml in SRC-fed C57BL/6J mice and was 6.7 ± 0.8 pg/ml in HFD- fed KKAy mice (*P* < 0.01) vs 3.2 ± 0.4 pg/ml in SRC-fed KKAy mice (Figure 4C), indicating a role of overweight- or obesity-associated inflammation in carcinogenesis. Additionally, serum 8-OHdG was 7.2 ± 0.8 ng/ml in HFD- fed C57BL/6J mice (*P* < 0.01) vs 4.5 ± 0.4 ng/ml in SRC-fed C57BL/6J mice and was 8.5 ± 0.9 ng/ml in HFD- fed KKAy mice (*P* < 0.01) vs 5.2 ± 0.5 ng/ml in SRC-fed KKAy mice (Figure 4D). Serum ROS was 85.4 ± 8.7 U/ml in HFD- fed C57BL/6J mice (*P* < 0.01) vs 51.3 ± 4.6 U/ml in SRC-fed C57BL/6J mice and was 96.9 ± 10.2 U/ml in HFD- fed KKAy mice (*P* < 0.01) vs 58.1 ± 4.8 U/ml in SRC-fed KKAy mice (Figure 4E), also demonstrating increased oxidative stress. These results were further corroborated by a higher plasma level of the vascular disruption marker 5-HIAA (286.7 ± 31.4 nmol/ml in HFD- fed C57BL/6J mice (*P* < 0.01) vs 195.4 ± 22.3 nmol/ml in SRC-fed C57BL/6J mice and was 321.6 ± 35.8 nmol/ml in HFD- fed KKAy mice (*P* < 0.01) vs 218.5 ± 24.2 nmol/ml in SRC-fed KKAy mice, Figure 4F), decreased lung epithelial integrity (Evans blue exudation 0.513 ± 0.054 in HFD- fed C57BL/6J mice (*P* < 0.01) vs 0.356 ± 0.042 in SRC-fed C57BL/6J mice and was 0.538 ± 0.055 in HFD- fed KKAy mice (*P* < 0.01) vs 0.373 ± 0.044 in SRC-fed KKAy mice, Figure 4G) and increased lung water content (was 291.6 ± 24.7 mg in HFD- fed C57BL/6J mice (*P* < 0.01) vs 245.3 ± 23.2 mg in SRC-fed C57BL/6J mice and was 372.8 ± 32.5 mg in HFD- fed KKAy mice (*P* < 0.01) vs 317.6 ± 28.6 mg in SRC-fed KKAy mice, Figure 4H).

### HFD-induced overweight or obesity increases cellular signaling molecules

**Figure 5:**
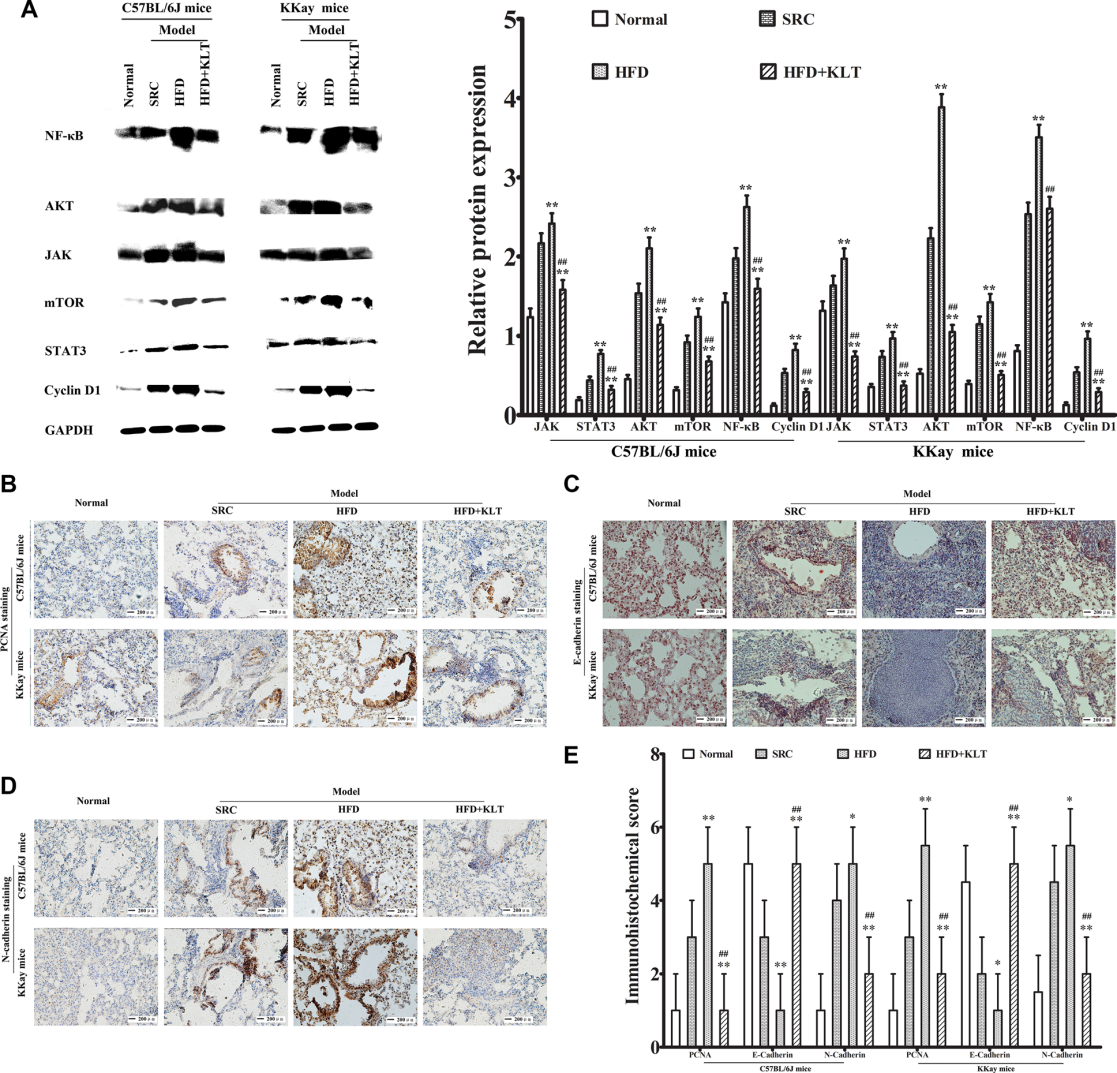
HFD-induced overweight or obesity increases cellular signaling molecules, lung epithelial proliferation and the EMT (**A**) Cellular signaling molecules including JAK, STAT3, Akt, mTOR, NF-κB and cyclin D1, as measured by Western blotting. (**B**) PCNA staining, (**C**) E-cadherin staining, (**D**) N-cadherin staining., as determined by immunohistochemistry. (**E**) Immunohistochemical score. Images were captured at ×40 magnification. The data are presented as the mean ± SD of triplicate assays, and statistical significance was determined by ANOVA, and unpaired, 2-tailed Student's *t* test was used to compare difference between two groups. **vs SRC or ^##^*vs* HFD; *P* < 0.01. HFD, high-fat diet; SRC, standard rodent chow; KLTI, kanglaite injection. Normal was defined as the non-carcinogenic mice fed in parallel.

Signaling mechanisms control cellular functions including cell survival, proliferation and growth [19]. Alterations in signal transduction cascades resulting from gene mutation or micro-environmental factors contribute to the development of cancer [20]. To further understand why obesity or overweight increases lung carcinogenesis, we examined overweight- or obesity-associated changes in cellular signaling molecules, including janus kinase (JAK), signal transducers and activator of transcription 3 (STAT3), protein kinase B (Akt), mammalian target of rapamycin (mTOR), nuclear factor-κB (NF-κB) and cyclin D1 at 21 week point after induction in a urethane-induced lung carcinogenic model by Western blot analyses. As shown in Figure 5A, overweight and obesity significantly increased the relative lung protein expression of JAK (2.415 ± 0.133 in HFD-fed C57BL/6J mice *vs* 2.167 ± 0.125 in SRC-fed C57BL/6J mice (*P* < 0.01) and 1.972 ± 0.131 in HFD-fed KKAy mice *vs* 1.634 ± 0.123 in SRC-fed KKAy mice (*P* < 0.01)), STAT3 (0.768 ± 0.052 in HFD-fed C57BL/6J mice *vs* 0.436 ± 0.051 in SRC-fed C57BL/6J mice (*P* < 0.01) and 0.963 ± 0.085 in HFD-fed KKAy mice *vs* 0.735 ± 0.074 in SRC-fed KKAy mice (*P* < 0.01)), Akt (2.102 ± 0.138 in HFD-fed C57BL/6J mice *vs* 1.535 ± 0.122 in SRC-fed C57BL/6J mice (*P* < 0.01) and 3.882 ± 0.169 in HFD-fed KKAy mice *vs* 2.226 ± 0.132 in SRC-fed KKAy mice (*P* < 0.01)) and mTOR (1.237 ± 0.106 in HFD-fed C57BL/6J mice *vs* 0.918 ± 0.085 in SRC-fed C57BL/6J mice (*P* < 0.01) and 1.423 ± 0.108 in HFD-fed KKAy mice *vs* 1.144 ± 0.098 in SRC-fed KKAy mice (*P* < 0.01)). Similarly, the lung NF-κB levels (2.624 ± 0.145 in HFD-fed C57BL/6J mice *vs* 1.976 ± 0.131 in SRC-fed C57BL/6J mice (*P* < 0.01) and 3.504 ± 0.158 in HFD-fed KKAy mice *vs* 2.535 ± 0.14 in SRC-fed KKAy mice (*P* < 0.01)) was directly correlated with increased JAK, STAT3, Akt and mTOR levels in HFD-fed mice. Specifically, the relative protein expressions of lung cyclin D1 (0.818 ± 0.084 in HFD-fed C57BL/6J mice *vs* 0.528 ± 0.055 in SRC-fed C57BL/6J mice (*P* < 0.01) and 0.961 ± 0.093 in HFD-fed KKAy mice *vs* 0.538 ± 0.066 in SRC-fed KKAy mice (*P* < 0.01)) was significantly increased in HFD-fed mice, indicating overweight- or obesity-associated changes in cellular signaling.

### HFD-induced overweight or obesity increases lung epithelial proliferation and the EMT

Carcinogenesis is the *final result of* epithelial proliferation and the epithelial- mesenchymal transition (EMT) [21]. To verify that overweight or obesity plays a role in carcinogenesis, we examined overweight- or obesity-associated changes in lung epithelial proliferation and the EMT by immunohistochemical analysis at 21 week point after induction in a urethane-induced lung carcinogenic model. As shown in Figure 5B–5E, significantly higher levels of the lung epidermal proliferation marker PCNA (score 5 ± 1 in HFD-fed C57BL/6J mice *vs* 3 ± 1 in SRC-fed C57BL/6J mice (*P* < 0.01) and 5.5 ± 1 in HFD-fed KKAy mice *vs* 3 ± 1 in SRC-fed KKAy mice (*P* < 0.01)), significantly induction of the lung EMT as indicated by the decreased E-cadherin levels (score 1 ± 1 in HFD-fed C57BL/6J mice *vs* 3 ± 1 in SRC-fed C57BL/6J mice (*P* < 0.01) and 1 ± 1 in HFD-fed KKAy mice *vs* 2 ± 1 in SRC-fed KKAy mice (*P* < 0.05)), and the increased N-cadherin levels (score 5 ± 1 in HFD-fed C57BL/6J mice *vs* 4 ± 1 in SRC-fed C57BL/6J mice(*P* < 0.05) and 5.5±1 in HFD-fed KKAy mice *vs* 4.5 ± 1 in SRC-fed KKAy mice (*P* < 0.05)) were detected in the overweight and obese mice. These results suggest that overweight and obesity promoted lung epithelial proliferation and the EMT.

### KLTI reduces susceptibility to overweight or obesity -promoted lung cancer progression

To confirm whether overweight or obesity is a suitable drug target for KLTI, mice were fed either the SRC or HFD and were orally administered KLTI once daily for 20 weeks. Under these experimental conditions, chronic KLTI treatment did not affect the body weights of the SRC-fed mice (BW 31.4 ± 2.2 g in SRC-fed C57BL/6J mice *vs* 31.3 ± 2.3 g in SRC+KLTI-fed C57BL/6J mice (*P* > 0.05) and 45.6 ± 2.8 g in SRC-fed KKAy mice *vs* 44.5 ± 3.1 g in SRC+KLTI-fed KKAy mice (*P* > 0.05)). In contrast, this treatment significantly attenuated weight gain in the HFD-fed mice (BW 35.5 ± 2.3 g in HFD-fed C57BL/6J mice *vs* 32.1 ± 2.4 g in HFD+KLTI-fed C57BL/6J mice (*P* < 0.05) and 56.7 ± 3.1 g in HFD-fed KKAy mice *vs* 48.5 ± 3.2 g in HFD+KLTI-fed KKAy mice (*P* < 0.01)) (Figure 1A) without affecting the food intake (data not shown). KLTI had a smaller but still significant effect on the lung carcinoma incidence and multiplicity in the two strains of mice fed the SRC compared with those fed the HFD in a urethane-induced lung carcinogenic model (Figure 1B and 1C, *P* < 0.05). However, KLTI did not significantly affect tumor size (Figure 2A, *P* > 0.05), lung metastasis (Figure 2B, *P* > 0.05) or cancer-related survival (Figure 2C, *P* > 0.05) in the two SRC-fed strains of LLC allograft model mice. In contrast, KLTI significantly decreased tumor development in the two HFD-fed strains of mice, as indicated by the decreases in tumor size (Figure 2A, *P* < 0.01) and lung metastasis (Figure 2B, *P* < 0.01), and increased cancer-related survival (Figure 2C, *P* < 0.01).

Consistent with the decreased HFD-induced weight gain, chronic KLTI treatment reversed the obesity- or overweight-induced decrease in glucose tolerance (Figure 3A, *P* < 0.01), insulin tolerance (Figure 3B, *P* < 0.01) and lung epithelial integrity (Figure 4G, *P* < 0.01) and increased the serum levels of insulin (Figure 3C, *P* < 0.01), adiponectin (Figure 3D, *P* < 0.01), leptin (Figure 3E, *P* < 0.01) and cholesterol (Figure 3F, *P* < 0.01) as well as those of the inflammation markers IL-6 (Figure 4A, *P* < 0.01), hs-CRP (Figure 4B, *P* < 0.01) and TNF-α (Figure 4C, *P* < 0.01), the oxidative stress markers 8-OHdG (Figure 4D, *P* < 0.01) and ROS (Figure 4E, *P* < 0.01), the vascular disruption marker plasma 5-HIAA (Figure 4F, *P* < 0.01), and the lung water content (Figure 4H, *P* < 0.01) in the two strains of mice fed the HFD.

To clarify the specific role of KLTI in overweight- or obesity-related carcinogenesis, growth factor signaling, lung epithelial proliferation, EMT and stem cell activation assays were also performed. Western blot analyses revealed that KLTI decreased cellular signaling molecules including JAK (*P* < 0.01), STAT3 (*P* < 0.01), Akt (*P* < 0.01), mTOR (*P* < 0.01), NF-κB (*P* < 0.01) and cyclin D1 (*P* < 0.01) in the two strains of KLTI-treated mice fed HFD (Figure 5A). Immunohistochemical analyses demonstrated that the level of the lung epithelial proliferation marker PCNA was significantly reduced (Figure 5B and 5E, *P* < 0.01) and that the EMT was significantly suppressed, as indicated by the decreased E-cadherin level (Figure 5C and 5E, *P* < 0.01) and the increased N-cadherin level (Figure 5D and 5E, *P* <0.01) in the KLTI-treated mice. These results indicate that the KLTI treatment protected mice from HFD-induced overweight and obesity and subsequently reduced their susceptibility to metabolic dysfunction-related lung carcinogenesis.

### KLTI induces cell functional changes *in vitro*

**Figure 6:**
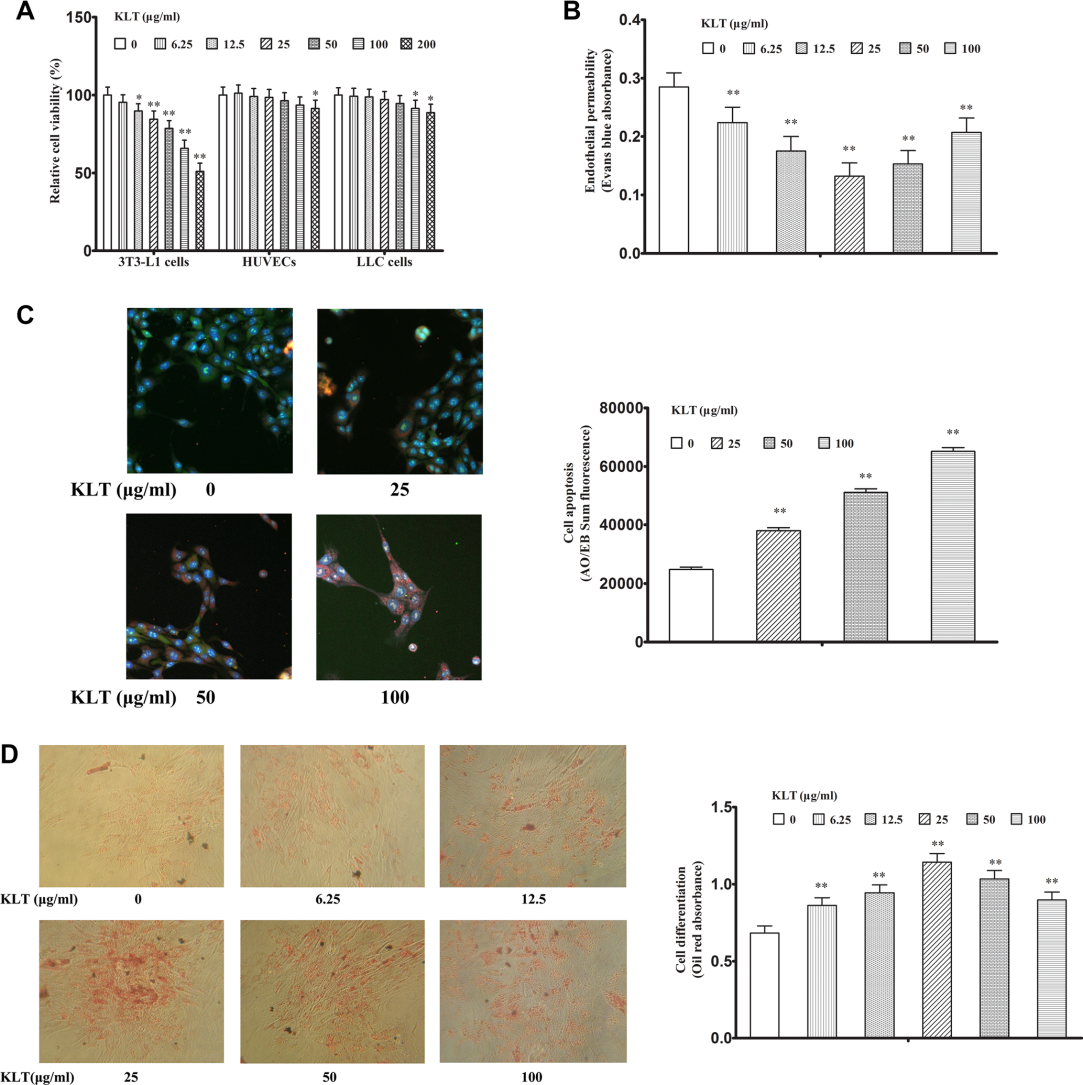
KLTI induces cell functional changes *in vitro* KLTI (**A**) Relative cell viability, as examined by MTT assay. (**B**) Endothelial permeability of HUVECs. (**C**) 3T3-L1 preadipocyte apoptosis, as determined by AO/EB staining. as (**D**) 3T3-L1 preadipocyte differentiation, as determined by Oil red staining. The data are presented as the mean ± SD of triplicate assays, and statistical significance was determined by ANOVA, and unpaired, 2-tailed Student's *t* test was used to compare difference between two groups. *P < 0.05; **P < 0.01 *vs* KLTI-untreated cells (0 concentration). KLTI, kanglaite injection.

KLTI is a molecularly targeted agent that is prepared as a microemulsion for use in cancer therapy, and it has inhibitory effects on many tumor types [10]. To examine how KLTI affects cell function, we selected Mouse LLC cells which represents lung cancer cells, HUVECs which represents endothelial cell and mouse pre-adipocytes (3T3-L1) which represents adipocytes to conduct assays of cell proliferation, endothelial permeability and differentiation. We found that KLTI at a concentration of less than 200 μg/ml significantly suppressed the proliferation of 3T3-L1 preadipocyte cells in a dose-dependent manner but had little effect on HUVEC and LLC cell proliferation (Figure 6A). We also found that KLTI treatment prevented the increase in endothelial cell permeability caused by the adipocyte-conditioned media in HUVECs (Figure 6B). Finally, we examined the effect of KLTI on cell apoptosis and differentiation in 3T3-L1 preadipocytes and found that it increased cell apoptosis at concentrations ranging from 25–100 μg/ml and induced differentiation at concentrations ranging from 6.25–100 μg/ml in 3T3-L1 preadipocytes (Figure 6C and 6D).

## DISCUSSION

Although several potential mechanisms linking obesity and cancers have been proposed [22], no conclusive evidence exists so far with regards to the roles of obesity and its related metabolic abnormalities in lung cancer progression. In this study, we evaluated the effects of HFD-induced overweight or obesity on lung carcinoma initiation and progression using an urethane-induced lung cancer model and a LLC allograft model. We show that HFD-induced overweight or obesity promoted lung carcinoma formation and progression in these animal models. Moreover, HFD-induced overweight or obesity leads to energy imbalance, decreased levels of cellular signaling molecules and increased lung epithelial proliferation and the EMT. More importantly, KLTI reduces susceptibility to overweight or obesity-promoted lung cancer progression *in vivo* and induces cell functional changes *in vitro*. Here, we not only identified a potential relationship between obesity and lung cancer progression but also provided a strategy for lung cancer prevention.

KLTI, composed of seven types of fatty acids that is extracted from Coix seed, has been demonstrated to be an effective anticancer treatment. Considering that it is from a nutritious food Adlay seed that has no overtly toxic effects and is edible, KLTI was administered by gavage to avoid tumor targeting effect and to assess its preventive effect on overweight or obesity-related pro-carcinogenesis, regardless of drug metabolism. However, the development of preventive agents has unique challenges and special considerations, and it does not differ significantly from the development of therapies for advanced cancers [23]. In this study, we first examined whether overweight or obesity increases the lung carcinoma incidence in a mouse-urethane lung carcinogenic model. We found that HFD-induced overweight in C57BL/6J mice and obesity in KKAy mice significantly increased the lung carcinoma incidence and multiplicity, indicating a potential relationship between obesity and lung cancer. This result was further confirmed in an LLC allograft model, in which overweight and obesity were found to be associated with increased tumor size, to promote lung metastasis and to decrease cancer-related survival. In addition, obesity and overweight increased inflammation and oxidative stress, altered the levels of JAK, STAT3, Akt, mTOR, NF-κB and cyclin D1, and enhanced lung epithelial proliferation and the EMT, thereby demonstrating plausible links between positive energy balance states and lung cancer promotion. Finally, we treated HFD-fed mice with KLTI and found that it significantly decreased lung carcinogenesis and tumor development in the two strains of mice but had reduced or little efficacy in the SRC-fed mice. With regard to results that KLTI did not significantly affect tumor size, lung metastasis or cancer-related survival in the two SRC-fed strains of mice receiving the SRC in the LLC allograft model mice, we believe that the preventive effect of KLTI on overweight and obesity-related pro-cancer is one of its therapeutic characteristic. The therapeutic characteristic of KLTI in adipocytes was further confirmed by the findings that KLTI suppressed cell proliferation and induced cell apoptosis and differentiation in 3T3-L1 preadipocyte cells but had few effects on HUVECs and LLC cell proliferation. Yet, there still remains the possibility that under a non-lethal dosage, KLTI is able to affect the function of HUVECs or LLC cells, such as reduction of endothelial cell permeability and suppression of LLC spontaneous metastasis in this study, which may explain why KLTI still reduced the lung cancer development in urethane-induced lung cancer mice fed the SRC. Simultaneously, it also suggests that KLTI is suitable for obesity-related cancer prevention but not thiner cancer patients.

The relationship between cancer and metabolic disorders was recognized several decades ago, but the underlying mechanisms involved in cancer development and progression remain obscure [24]. We has revealed that tissue interstitial fluid (TIF) provides better nutrition to tumors than angiogenesis and that it promotes the development of malignant phenotypes of lung cancer independently of angiogenesis [25]. In this study, we found that overweight or obesity decreased lung epithelial integrity, promoted vascular disruption and increased the lung water content. Considering our finding that KLTI did not significantly affect on tumor size, lung metastasis or cancer-related survival in the two SRC-fed strains of LLC allograft model mice and the fact that KLTI alone cannot cure lethal human malignancies [13], we believe that the pro-TIF effect of overweight and obesity is one of the main mechanisms underlying the increase in cancer-related *mortality,* and we support the use of Coix seed as a candidate strategy for preventing obesity-related carcinogenesis and cancer edema-related *mortality*.

An understanding of the interplay between local and systemic alterations involved in the obesity-cancer link provides the basis for developing interventions aimed at mitigating the protumorigenic effects [26]. Most studies support a relationship between body adiposity and site-specific mortality or cancer progression [27]. However, most of the research was not specifically designed to study these outcomes and, therefore, several methodological issues should be considered before integrating their results to draw conclusions [28]. Our results provide new insights into the role of HFD-induced obesity in lung cancer and demonstrate that overweight and obesity can increase lung cancer formation. This is the first study to show that obesity can directly accelerate carcinogen-induced lung cancer progression and oral KLTI can decrease the pro-carcinogenic effect of HFD-induced overweight or obesity. Many researchers have proposed the concept of “survival with cancer” and insist that controlling cancer and causing cancer cells to “remain static” and to “hibernate” for a long period time is better than attempting to reduce the tumor volume or completely kill all cancer cells [29]. Based on this view, we believe that KLTI Coix seed oil possesses advantages as an adjuvant therapy for alleviating the symptoms of terminal-stage cancer, for which no further treatment options can be offered in Western medicine.

## MATERIALS AND METHODS

### Animals

Cohorts of 5- to 6-week old female C57BL/6J mice and KKAy mice were obtained from Beijing Weitong Lihua Animal Co. All mice were housed in individual ventilated cages under a 12-h light-dark cycle (lights on from 7:00 AM to 7:00 PM). The animals were fed aSRC (Henan Provincial Medical Laboratory Animal Center) or a HFD (Beijing Weitong Lihua Animal Co.) and water. All animal procedures used in this study were approved by the Animal Experimentation Ethics Committee (AEEC) of Henan University (permission number HUSAM2014-216), and all procedures were performed in strict accordance with the Guide for the Care and Use of Laboratory Animals and the regulations of the animal protection committee to minimize suffering and injury. The animals were monitored daily and euthanized humanely by an overdose of carbon dioxide (CO_2_) at the end of the experiment or at the first sign of shortness of breath, reduced locomotion and reduced body weight (greater than 20% of the total body weight). All surgeries were performed under general anesthesia (intraperitoneal injection of 45 mg/kg pentobarbital sodium, R&D Systems, M→N, USA), and all efforts were taken to minimize suffering.

### Urethane-induced lung adenocarcinoma model

C57BL/6J or KKAy mice were administered freshly prepared urethane to induce lung adenocarcinoma according to our previously published protocol [17]. The mice received intraperitoneal injection of urethane (U2500, Sigma, St. Louis, MO, USA) (600 mg/kg body weight, dissolved in sterile 0.9% NaCl) once weekly for 10 weeks. Following urethane injection, the mice were fed the SRC or HFD (60% kcal from fat) and received 20 ml/kg KLTI (containing 0.1 g Coix seed oil per ml for injection) by *gavage* once daily for 20 weeks. During the study, food and water were provided *ad libitum*. The health of the mice was monitored daily, and their body weights were measured weekly. At 21 weeks after the first urethane injection, orbital venous blood was collected for use in a plasma 5-HIAA (a vascular disruption marker) assay performed using an ELISA kit (R&D Systems, MN, USA). Finally, the mice were euthanized, the lung carcinoma incidence and multiplicity (average number of carcinomas per mouse) were determined, and the lungs were biochemically processed.

Each group contained twenty-two mice in experiments, and a normal group was included that contained non-cancerous mice fed in parallel. At the end of the experiment, each group contained at least twenty mice. In all experimental procedures, one to two mice in each group exhibited clear evidence of myocardial infarction and were excluded from the study and humanely euthanized.

### Insulin and glucose tolerance tests

Two tests were carried out at 1 week before the mice were euthanized. For the insulin tolerance test, the mice were fasted for 6 h and were then intraperitoneally injected with 1 U/kg BW insulin (Sigma, St. Louis, MO). For the glucose tolerance test, the mice were fasted for 16 h and were then intraperitoneally injected with 2 g/kg BW glucose (Sigma, St. Louis, MO, USA). Blood was collected from the tail veins of the mice at 0, 15, 30, 60, and 90 min after injection, and the glucose concentrations were analyzed using a Glucometer (Bayer, Pittsburgh, PA).

### Clinical chemistry tests

Blood samples were collected from the orbital venous plexus at death after 6 h of fasting for chemical analyses. The serum concentrations of insulin, adiponectin (R&D Systems, MN, USA), leptin (R&D Systems, MN, USA), cholesterol (Sigma, St. Louis, MO, USA), IL-6 (R&D Systems, MN, USA), hs-CRP (R&D Systems, MN, USA) and TNF-α (R&D Systems, MN, USA) were determined according to the manufacturer's protocols.

### Oxidative stress analysis

8-OHdG (R&D Systems, MN, USA) levels were measured using an ELISA assay kit. Serum levels of ROS (Nanjing Jiancheng Bioengineering Institute, China), a marker of oxidative stress, were also determined using an ELISA assay kit.

### Lung epithelial integrity and lung water content measurements

The integrity of the lung epithelium was assayed using the Evans blue extra-barrier technique as previously described [29], and lung water content was measured as previously described [25].

### Immunohistochemistry

For immunohistochemical (IHC) analyses, serial lung sections (5 μm) were cut and attached to Superfrost Plus microscope slides. The slides were warmed to 60°C for 1 h, deparaffinized in xylene and rehydrated in decreasing concentrations of ethanol. After being blocked with 3% hydrogen peroxide for 10 min, the sections were incubated with 0.1% Triton X-100 in PBS for 20 min at room temperature. Then, they were immersed in a blocking solution containing 5% BSA for 20 min at room temperature. Next, the sections were incubated with primary antibodies (1:100) against PCNA (ProteinTech Group, Wuhan, China), E-cadherin (ProteinTech Group, Wuhan, China) and N-cadherin (ProteinTech Group, Wuhan, China) overnight at 4°C. Antigen–antibody binding was detected using a Dako Cytomation LSAB System-HRP Kit (Dako Cytomation, Carpinteria, CA, USA). Signal was developed using the peroxidase substrate DAB (Nanjing Jiancheng Bioengineering Institute, Chinawhich, yields a brown reaction product) or using AEC (Nanjing Jiancheng Bioengineering Institute, China, yields a red reaction product). All sections were counterstained with hematoxylin and imaged under a microscope. Semi-quantitative histological scores (HSCORE) wwere calculated, as described previously [29].

### Western blot analysis

Lung extracts were prepared in radioimmunoprecipitation assay (RIPA) cell lysis buffer (Solarbio, Beijing, China), and equal amounts of protein were separated on a 12% sodium dodecyl sulfate–polyacrylamide electrophoresis gel, electroblotted on nitrocellulose membranes, and probed with specific antibodies against JAK (ProteinTech Group, Wuhan, China, 1:1000), STAT3 (ProteinTech Group, Wuhan, China, 1:2000), Akt (ProteinTech Group, Wuhan, China, 1:1000), mTOR (ProteinTech Group, Wuhan, China, 1:1000), NF-κB (ProteinTech Group, Wuhan, China, 1:1000), cyclin D1 (ProteinTech Group, Wuhan, China, 1:2000) and GAPDH (ProteinTech Group, Wuhan, China, 1:2000). The secondary antibody used was Peroxidase-conjugated Affinipure Goat anti-rabbit IgG (H + L) (ProteinTech Group, 1:5000). Antibody binding was detected by enhanced chemiluminescence according to the manufacturer's protocol (Pierce, Rockford, IL). Band densities were quantified using Image software (NIH, Bethesda, MD, USA) and normalized according to the reference gene GAPDH. All values were then normalized to relative GAPDH expression.

### Tumor allograft experiment

LLC cells were maintained in RPMI 1640 medium containing 10% FBS until they reached 80% confluence. Then, the cells were collected, and 200 μL saline containing 1 × 10^6^ LLC cells was injected subcutaneously into the lateral axilla of normal C57BL/6J mice or KKAy mice, as well as into those of HFD-fed lean C57BL/6J mice or obese KKAy mice. Following tumor inoculation, the mice were administered 20 ml/kg KLTI by *gavage* once daily for four weeks. At 28 days after tumor incubation, the tumors were dissected and weighed, and the number of tumors on the lung surface was counted.

Ten mice in each group were used for survival analysis until all of the mice died.

### Cell culture

Mouse LLC cells, HUVECs and mouse pre-adipocytes (3T3-L1) were purchased from the Cell Bank of the Chinese Academy of Sciences in Shanghai. LLC cells were grown in RPMI 1640 medium (Sigma, St. Louis, MO, USA) supplemented with 10% (v/v) fetal bovine serum (FBS, Gibco Invitrogen, Carlsbad, CA). HUVECs were cultured in DMEM/F12 medium (Invitrogen, USA) supplemented with 10% (v/v) FBS. 3T3-L1 preadipocytes were cultured in DMEM medium (Sigma, St Louis, MO, USA) supplemented with 10% (v/v) newborn calf serum (NCS, Gibco Invitrogen, Carlsbad, CA). All cells were grown in a humidified atmosphere with 5% CO2 and 95% air at 37°C.

### Cell proliferation assay

3T3-L1 preadipocyte cells and HUVECs were seeded at 10,000 cells per well and LLC cells were seeded at 5,000 cells per well in 96-well plates overnight at 37°C. Cells were treated with the indicated concentrations of KLTI, and each condition was tested using six replicates. During the final 4 h of the 48-h incubation period, the supernatants were discarded, and 100 μL MTT (Solarbio, Beijing, China) (0.5 mg/ml) was added to each well. After 4 h, the MTT was discarded, and 100 μL DMSO (Solarbio, Beijing, China) was added to each well. The absorbance of each sample was measured at 570 nm using a plate reader (Elx-800; Bio-Tek, USA). The results of the cell proliferation assay are presented as a percentage of the control cells. All experiments were repeated in triplicate.

### Adipocyte differentiation

3T3-L1 preadipocyte cells were cultured in DMEM complete medium and grown to confluence. The medium was replaced with complete medium containing 1.7 μM insulin, 1.0 μM dexamethasone and 0.5 mM 3-isobutyl-1-methylxanthine (IBMX) (Solarbio, Beijing, China), and the cells were cultured for 48 h. Then, the medium was replaced with complete medium containing 1.7 μM insulin (Sigma, St. Louis, MO, USA) and cultured for an additional 48 h. Finally, the cells were maintained in complete medium containing the indicated concentrations of KLTI as Coix seed oil, and the medium was replaced with fresh medium containing the indicated concentrations of KLTI every 48 h. On day 8, the supernatant was collected and filtered to remove debris to obtain adipocyte-conditioned medium. The cells were washed twice with cold PBS (136.9 mM NaCl, 2.68 mM KCl,4 mM Na2HPO4, and 1.76 mM KH2PO4; pH 7.4), fixed in 3.7% paraformaldehyde (Solarbio, Beijing, China) for 1 h and then stained with 0.2% (w/v) Oil red O(Rowley Biochemical, USA) in a 60% isopropanol (IT7048, Shanghai Sangon Biological Engineering Co. Ltd, China) solution for 1 h. Oil red staining was observed and analyzed using an inverted microscope (Olympus, Tokyo, Japan). Stained cells were treated with 100% isopropyl alcohol to extract the Oil red O, and the colorimetric intensity of the extract was measured at 595 nm to determine the lipid content.

### *In vitro* permeability assay

Endothelial cell permeability was quantified by spectrophotometric measurement of the flux of Evans blue-bound albumin (Solarbio, Beijing, China) across functional cell monolayers using a modified two-compartment chamber model. Briefly, HUVECs were plated (5 × 10^4^ cells/well) in a 3-μm-pore size, 12-mm-diameter Transwell apparatus for 3 days. Confluent monolayers were incubated in medium supplemented with 10% adipocyte-conditioned medium for 24 h. Then, the transwell inserts were washed with PBS, and 0.5 ml Evans blue (0.67 mg/ml) diluted in growth medium containing 4% bovine serum albumin (BSA) (Life Technologies, New York, USA) was added. Fresh growth medium was added to the lower chamber, and the medium in the upper chamber was replaced with Evans blue/BSA. Ten minutes later, the optical density of the solution in the lower chamber was measured at 650 nm.

### Apoptosis detection

Apoptosis was evaluated by acridine orange (AO) / ethidium bromide (EB) (Sigma, St. Louis, MO, USA) fluorescence staining. Briefly, 4 μL of dye mixture (100 μg/mL AO and 100 μg/mL EB in PBS) were added to a suspension of KLTI-treated LLC cells cultured under serum-free conditions for 12 h, and the cells were washed once with PBS. After staining, the cells were visualized and analyzed using an IN Cell Analyzer 2000 (GE Healthcare).

### Statistical analyses

The Shapiro–Wilk test was conducted to test for normal distribution. Normally distributed data are presented as the mean ± SD and were statistically analyzed using GraphPad Prism, Version 5.0 (San Diego, CA, USA). Differences among the groups were evaluated by ANOVA one-way or two-way analysis. A *P* value of less than 0.05 was considered statistically significant.

## Abbreviations

5-HIAA: 5-hydroxy indole acetic acid
8-OHdG: 8-hydroxy-2′-deoxyguanosine
Akt: protein kinase B
AO: acridine orange
BSA: bovine serum albumin
EB: ethidium bromide
EMT: epithelial-mesenchymal transition
FBS: fetal bovine serum albumin
GAPDH: glyceraldehyde-3-phosphate dehydrogenase
HFD: high-fat diet
HRP: horseradish peroxidase
HSCORE: histological score
hs-CRP: high-sensitivity C-reactive protein
HUVEC: human umbilical vein endothelial cell
IHC: immunohisochemical
JAK: janus kinase
KLTI: kanglaite injection
mTOR: mammalian target of rapamycin
NF-κB: nuclear facter-κB
NSCLC: non-small cell lung cancer
PBS: phosphate buffer saline
PCNA: proliferating cell nuclear antigen
ROS: reactive oxygen species
SRC: standard rodent chow
STAT3: Signal Transducers and Activator of Transcription 3
TCM: traditional Chinese medicine
TIF: tissue interstitial fluid
TNF-α: tumor necrosis factor α
